# Noise in the Sea and Its Impacts on Marine Organisms

**DOI:** 10.3390/ijerph121012304

**Published:** 2015-09-30

**Authors:** Chao Peng, Xinguo Zhao, Guangxu Liu

**Affiliations:** College of Animal Sciences, Zhejiang University, Hangzhou 310058, Zhejiang, China; E-Mails: SUPERJUNIOR_PC@163.COM (C.P.); xinguozhao@zju.edu.cn (X.Z.)

**Keywords:** noise, marine organisms, auditory masking, behavior alteration, metabolism, recruitment, population composition

## Abstract

With the growing utilization and exploration of the ocean, anthropogenic noise increases significantly and gives rise to a new kind of pollution: noise pollution. In this review, the source and the characteristics of noise in the sea, the significance of sound to marine organisms, and the impacts of noise on marine organisms are summarized. In general, the studies about the impact of noise on marine organisms are mainly on adult fish and mammals, which account for more than 50% and 20% of all the cases reported. Studies showed that anthropogenic noise can cause auditory masking, leading to cochlear damage, changes in individual and social behavior, altered metabolisms, hampered population recruitment, and can subsequently affect the health and service functions of marine ecosystems. However, since different sampling methodologies and unstandarized measurements were used and the effects of noise on marine organisms are dependent on the characteristics of the species and noise investigated, it is difficult to compare the reported results. Moreover, the scarcity of studies carried out with other species and with larval or juvenile individuals severely constrains the present understanding of noise pollution. In addition, further studies are needed to reveal in detail the causes for the detected impacts.

## 1. Introduction

The French documentary *The Silent World*, co-directed by the famous French oceanographer Jacques-Yves Cousteau and director Louis Malle in 1956, presented a multi-colored and wonderful undersea world full of life and energy that satisfied the curiosity of audiences at the time. However, with the deepening of the investigation of this “world,” the reality has proven not to be as silent as was initially thought. In fact, sound plays a vital role in the lives of many marine organisms in this undersea world.

Without doubt, anthropogenic sound from cargo ships, sonar, seismic testing, drilling, pile drivers, recreational holiday ships, and *etc.* has continued to grow in the last century [1,2,3,4,5]. Consequently, the level of underwater background noise worldwide has increased correspondingly due to increased anthropogenic activities, which gives rise to a new kind of pollution: noise pollution [6].

Noise may cause stress in animals, increase the risk of mortality by unbalancing predator-prey interaction, and interfere with sound-based orientation and communication, especially in reproductive contexts [7]. There is growing international concern regarding the impact of anthropogenic noise on marine organisms [8]. A number of studies have shown that the effects of anthropogenic sound on marine organisms can range from no influence to immediate death depending on the differences in the intensity and frequency of the noise and the distance from the noise source. However, the mechanisms underlying these effects are still poorly understood [3,9,10,11].

In this paper, we summarize (1) the sources of biotic and abiotic sound undersea and the characters of anthropogenic sound, (2) the significance of sound to marine organisms, and (3) the effects of anthropogenic sound on various aspects of different species to elucidate the current understanding of noise pollution in the sea and the gaps in our current knowledge.

## 2. Sounds in the Sea and Their Biological Significances

### 2.1. Propagation of Sounds in the Sea

Knowledge of the feature of sound is essential to a full understanding of the impacts of sound on marine organisms. Frequency, wavelength, and intensity are the main parameters used to describe the characteristics of sound. One important feature of sound is its transmissibility in various mediums. Both the speed and the path upon which sound propagates are dependent on the characteristics of the medium through which it travelling. In the sea, variations in the properties of seawater such as temperature, pressure, and salinity all have significant effects on the speed of sound propagation [12]. As it travels through seawater with changing characteristics, the path that sound propagates changes as well due to refraction. The downward curving path found when sound travels in the thermocline is a typical example showing how the traveling path of sound is altered through refraction. As the sound travels downward in the thermocline, the water pressure increases gradually and leads to an upward alteration in the sound’s traveling path below the bottom of the thermocline. In this circumstance, theoretically the sound may travel at this depth, also known as “the deep sound channel,” without propagation loss [13].

Propagation loss is the reduction of sound intensity with propagation due to absorption and scattering. In the sea, propagation loss is dependent on a variety of variables, such as the distance away from the sound source, the location of sound source, the amount of particles suspended in the seawater, and the sound frequency. In the sea, the propagation of sound is also affected by reflection. Particularly when the water depth is less than the wavelength of the sound, a high propagation loss is expected [12].

### 2.2. Sources of Sounds in the Sea

The underwater environment consists of both biotic and abiotic sounds that are closely related to the survival and reproduction of marine organisms [11]. Biotic sound sources are produced by fish, invertebrates, marine mammals and other marine organisms, and are essential to communication, orientation, mate and prey detection, and echolocation [14,15]. Marine organisms can produce biotic sound in various ways [16]. Some organisms, such as cod (*Melanogrammus aeglefinus*, *Gadus morhua*, *Pollachius pollachius*, *Raniceps raninus*) can produce sound by vibrating their swim bladder through muscle strength [17]. Other species produce sound by rubbing hard parts of their body. For example, some catfishes (*Siluridae*) produce sound using their pectoral girdle, some cichlids (*Cichlidae*) create sound using their pharyngeal teeth, and the snapping shrimp (*Alpheus* spp. *and Synalpheus* spp.) produces mid-frequency sound (2 kHz–24 kHz) with its claws [3,15,18,19]. Abiotic sound sources, which provide important information about surrounding environments to marine organisms, can be divided into two categories: natural background sound and anthropogenic sound. Sea waves breaking on the coast, currents moving over reef, raindrops on the ocean surface, tides, oceanic turbulence and the sound produced by seaquakes and submarine volcano eruptions are typical natural background sounds [20,21].

The amount of variety of anthropogenic noises has risen significantly during the last few decades in both the open ocean and the highly-populated coastal areas due to increasing human activities [1,4,22,23]. Anthropogenic sounds emitted from different human activities vary significantly in terms of the frequencies and intensities (Table 1) [12,24,25,26]. Based on frequency and intensity characteristics, anthropogenic noise can be categorized into two main types: high-intensity impulsive noise and low-frequency stationary noise. High-intensity noise can be produced by pile driving, underwater blasting, seismic exploration and active sonar application [7]. Nowadays, pile driving, a construction activity, is predominantly found nearshore where the construction of bridges, ports, wind farms and other buildings occurs. Seismic exploration devices, mainly air guns, are used all over the world for undersea geological surveys and geophysical studies such as oil and gas exploration and seabed mapping [10]. Similarly, sonar generating noise at various intensities is widely used not only by navies but also by commercial ships, the fishing industry, and marine research organizations [10]. Low-frequency stationary noise can be generated by various ships and vessels [7]. Although the number of fishing vessels has not increased much since the 1960s, there are still about 1.2 million vessels in use [11]. In addition, the number of recreational boats has increased rapidly in coastal areas. Another growing source of marine low-frequency stationary noise is the proliferation of oceangoing freighters that transport large cargoes as a critical link for maintaining global commerce. The number of large cargo ships has steadily increased by 8%–14% in the first decade of 21 century [27].

**Table 1:** Examples of reported anthropogenic noise in the sea with various frequencies and intensity levels.

Types of the Anthropogenic Sound	Frequency	Intensity Level	References
Bottom-founded oil drilling and mining	4–38 Hz	119–127 dB re 1 μPa	Richardson *et al.*, 1995 [26]
Pile driving	30–40 Hz	131–135 dB re 1 μPa	Richardson *et al.*, 1995 [26]
Drillship	20–1000 Hz	174–185 dB re 1 μPa	Richardson *et al.*, 1995 [26]
Semisubmersible drilling vessel	10–4000 Hz	~154 dB re 1 μPa	Richardson *et al.*, 1995 [26]
Seismic airguns	100–250 Hz	240–250 dB re 1 μPa	Richardson *et al.*, 1995 [26]
The Acoustic Thermometry of Ocean Climate Project (ATOC)	~75 Hz	~195 dB re 1 μPa	Buck, 1995 [24]
Navy Sonar	100–500 Hz	~215 dB re 1 μPa	Conservation and development problem solving team, University of Maryland, 2000 [12]
High Frequency Marine Mammal Monitoring Sonar (HF/M3)	~3000 Hz	~220 dB re 1 μPa	Conservation and development problem solving team, University of Maryland, 2000 [12]
Supertanker & container ship	6.8–70 Hz	180–205 dB re 1 μPa	Richardson *et al.*, 1995 [26]; Gisiner *et al.*, 1998 [25]
Medium size ship (ferries)	~50 Hz	150–170 dB re 1 μPa	Richardson *et al.*, 1995 [26]
Boats (<30 m in length)	<300 Hz	~175 dB re 1 μPa	Richardson *et al.*, 1995 [26]
Small ship (support & supply ship)	20–1000 Hz	170–180 dB re 1 μPa	Richardson *et al.*, 1995 [26]

### 2.3. Biological Significances of Sound in the Sea

Weather variations (e.g., wind speed, the size of rain drops and the magnification of thunder), seasonal alternations, geological activities (e.g., volcanoes and earthquakes under the sea), the behavior of marine organisms, and human activities all contribute to ambient sound [3,21], which is crucial to marine organisms. Generally, marine organisms can obtain information from biotic and abiotic sound sources through their auditory systems and subsequently react correspondingly [3]. For instance, echolocation is applied via the auditory senses of many marine species and provides a 3D view of the surrounding environment for prey and predator detection [28,29,30,31]. In addition, species-specific sound bands were shown to be used by various marine organisms for attracting mates, aggregating, and engaging in territorial behavior [10,20,32,33].

## 3. The Effects of Anthropogenic Noise on Marine Organisms

Organisms vary in their absolute sensitivity and spectral range of hearing. Broadly, fishes can be categorized as hearing specialists (broad hearing frequency range with low auditory thresholds) or hearing generalists (narrow hearing frequency range with higher auditory thresholds) [11,34]. For instance, the Lusitanian toadfish (*Halobatrachus didactylus*), a hearing generalist, exhibits its best hearing sensitivity at sound frequencies between 50 Hz and 200 Hz. By contrast, the fathead minnow (*Pimephales promelas*), a hearing specialist, exhibits its most sensitive hearing range from 0.8 kHz to 2.0 kHz [35,36]. Similarly, in sea mammals, the fin whale (*Balaenoptera physalus*) has a wide hearing range from 0.01 kHz to 10 kHz, whereas the spectral range of hearing of a sea lion (*Zalophus californianus*) is relatively narrow, from 1kHz to 10 kHz [11]. The scope, intensity, and frequency of anthropogenic noise pollution may be much greater than natural acoustic stimuli and, therefore, this type of noise pollution has been shown to have a series of adverse influences on marine species [9]. Current knowledge indicates that anthropogenic noise can directly or indirectly affect many marine organisms, causing auditory masking [7], leading to cochlear damage [37], changing individual and/or social behavior [38], altering body metabolism [35], and hampering embryogenesis [39]. Therefore, noise pollution will not only pose a great threat to individual marine organisms but also may affect the composition, and subsequently the health and service functions of the ecosystem. For instance, some studies have shown that anthropogenic noise caused a reduction in the catch rate of some commercial marine species indicating a decrease in the service function of the ecosystem for providing fishery products [36,40,41,42,43].

### 3.1. Acoustic Masking and Physiological Damage to Hearing System

Many marine organisms depend on the interpretation of acoustic information of their surrounding environment for their survival. Thus, noise pollution can affect marine organisms’ acoustic communication through auditory masking (in which the perception of one sound is affected by the presence of another sound) and through physiological damage of hearing system (Table 2) [44].

Acoustic masking is considered to be one of the main effects of noise pollution on marine animals [7,45,46,47]. Southall *et al.* investigated the acoustic communication ranges of northern elephant seals (*Mirounga angustirostris*) and the results demonstrated that the varied communication ranges were partially dependent on the ambient noise conditions [45]. According to these results, anthropogenic noise might constrain acoustic communication of northern elephant seals through auditory masking. Codarin *et al.* pointed out that noise emanating from boating and shipping had a significant effect on three fish families, *Chromis chromis*, *Sciaena umbra*, and *Gobius cruentatus*, which have different hearing abilities [7]. The results showed that the noise emanating from a cabin cruiser substantially reduced the auditory sensitivity of these three fish families since their hearing thresholds during ambient noise played back in lab conditions were almost completely masked [7]. Similarly, it has been reported that only sound pressure above the normal hearing range can be perceived immediately after exposure to the noise stimulant in the harbor porpoise (*Phocoena phocoena*) and bottlenose dolphin (*Tursiops truncatus*) [48,49]. This phenomenon was also reported by Vasconcelos in the Lusitanian toadfish (*Halobatrachus didactylus*), in which study the signal had to be 36 dB louder to be perceived by the fish in the presence of ship noise [46]. A further comparison between the masked audiograms and the sound spectra of the toadfish’s mating and agonistic vocalizations showed that ship noise hampered the toadfish’s ability to perceive conspecific sounds [46]. Despite the paucity of direct experimental evidence in most marine species, it is highly likely that the auditory masking effects of the same noise differ among species as been reported in freshwater fishes [34,50,51].

Anthropogenic noise can lead to not only auditory masking but also to physiological damage in the hearing systems of marine animals. McCauley *et al.* found that the noise created by an operating air-gun severely damaged the ears of the pink snapper (*Pagrus auratus*), resulting in apparent ablated hair cells of the sensory epithelia [37]. Moreover, the damaged cochlear cells were not repaired or replaced until 58 days after exposure to the air-gun. Similarly, André *et al.* demonstrated morphological and ultrastructural evidence of massive acoustic trauma in four cephalopod species (*Loligo vulgaris*, *Sepia officinalis*, *Octopus vulgaris*, and *Illex coindetii*) subjected to low-frequency noise exposure, which caused permanent and substantial alterations of the sensory hair cells of the statocysts [52]. The noise generated by geophysical seismic surveys (peak sound pressure levels at 175 dB re 1 μPa) has been singled out as the cause for atypical mass strandings of giant squids (*Architeuthis dux*) as well [53]. The internal examinations showed that these stranded squids had suffered extensive damage to internal fibers and statocysts, their stomachs were ripped open, and their digestive tracts were mangled [53].

**Table 2:** Example studies showing effects of anthropogenic noise on acoustic communication and physiological hearing system of marine organisms.

Species	Types of Anthropogenic Noise	Effects	References
*M. angustirostris*	increased ambient noise	constrains acoustic communication	Southall *et al.*, 2003 [45]
*C. chromis*	boating and shipping noise	reduces auditory sensitivity and shifts the hearing threshold	Codarin *et al.*, 2009 [7]
*S. umbra*			
*G. cruentatus*			
*H. didactylus*	boating and shipping noise	constrains acoustic communication and shifts the hearing threshold	Vasconcelos *et al.*, 2007 [46]
*P. phocoena*	seismic air-gun shooting	shifts the hearing threshold	Lucke *et al.*, 2009 [48]
*T. truncatus*	experimental noise emanating device	shifts the hearing threshold	Nachtigall *et al.*, 2004 [49]
*P. auratus*	seismic air-gun shooting	damages the hearing sensory epithelia	McCauley *et al.*, 2003 [37]
*L. vulgaris*	experimental noise emanating device	damages the hearing sensory epithelia	André *et al.*, 2011 [52]
*S. officinalis*			
*O. vulgaris*			
*I. coindetii*			
*A. dux*	seismic air-gun shooting	damage to internal fibers, statocysts, stomachs, and digestive tracts	Guerra *et al.*, 2011 [53]

### 3.2. Behavior Alteration

In addition, anthropogenic noise can alter the individual behavior of some marine organisms, causing behavior alterations such as startle responses and attention distraction (Table 3). The behavioral responses of squid (*Sepioteuthis australis*) and two species of schooling demersal pelagic fish, trevally (*Pseudocaranx dentex*) and pink snapper (*P. auratus*) before, during, and after air-gun noise exposure were studied by Fewtrell and McCauley [54]. The results showed that fish responded to noise by moving to the bottom of the water column and swimming faster in more tightly cohesive groups, and both the fish and squid showed significant increases in their alarm responses. When exposed to naval mid-frequency sonar exercises, simulated military sonar, killer whale calls, or band-limited noise, disruption of foraging behavior and avoidance responses were found in the Blainville’s beaked whale, *Mesoplodon densirostris* [55]. Similarly, noise generated with seismic airgun (array peak source level 252 dB re 1 uPa) reportedly induced a dive response in loggerhead turtles, *Caretta caretta*, which indicated an induced avoidance response [56]. Schwarz and Greer also found that the net-penned Pacific herring (*Clupea pallasii*) reacted differently to various kinds of sounds [38]. The herring did not show a visible response to sonar, echo sounders, or any of the taped natural sounds, including rain on the water surface, gull (*Larus* spp.) cries, killer whale (*Orcinus orca*) vocalizations, barks of Steller sea lions (*Eumetopias jubatus*) and self-produced chirps and whistles. However, the sound of large vessels approaching at a constant speed and smaller vessels approaching at an accelerating speed led to avoidance responses in the herring, which indicated that herring are more sensitive to low frequency noise emitted from ships than high frequency sound from sonar and echo sounders [38]. Via underwater camera observation and time-budget analysis (time allocated to nest caring or inside of the shelters during the observation period), Picciulin *et al.* found that the playback of the recorded boat noises had no significant short-term behavioral effect while the time-budget analysis revealed a significant change in the total time spent in caring for their nests (*C. chromis*) or inside their shelters (*G. cruentatus*) [57]. Similarly, it has been demonstrated by Bruintjes and Radford that boat noise led to reduced nest digging, decreased defensive behavior against predators over eggs and fry, and increased aggression in a territorial and cooperatively breeding cichlid fish, *Neolamprologus pulcher* [58].

Kastelein *et al.* investigated the behavioral startle response thresholds of eight marine fish species including seabass (*Dicentrarchus labrax*), thicklip mullet (*Chelon labrosus*), pout (*Trisopterus luscus*), Atlantic cod (*G. morhua*), pollack (*P. pollachius*), horse mackerel (*Trachurus trachurus*), common eel (*Anguilla anguilla*), and Atlantic herring (*Clupea harengus*) exposed to 0.1–64 kHz noises [6]. The fish species were shown to respond differently to the tested noise, suggesting the reactions of fish species to noise were probably dependent on a complicated set of variables such as location, temperature, physiological state, age, body size, and school size [6]. Other than the startle response, an increase in food-handling error and a decrease in discrimination between food and non-food items were also detected when captive three-spined sticklebacks (*Gasterosteus aculeatus*) were exposed to brief and prolonged experimental noise respectively, suggesting a shift in attention and foraging efficiency caused by noise [59].

Besides the increased avoidance behavior, being distracted by noise also made some marine species more vulnerable to predation. When exposed to boat motor playback, Caribbean hermit crabs (*Coenobita clypeatus*) allowed a simulated predator to approach closer than usual before the hiding response was activated [60]. Similarly, a slower retreat-to-shelter behavior after detection of a simulated predator attack, besides the disruption of foraging and a turn-upside-down behavior, were found in the shore crab (*Carcinus maenas*) when exposed to a ship noise playback [61].

A method of coping with the acoustic masking effect of noise, modified sound-producing behavior was found in humpbacks (*Megaptera novaeangliae*), bottlenose dolphins (*T. truncatus*), North Atlantic right whales (*Eubalaena glacialis*), and South Atlantic right whales (*E. australis*) [62,63,64]. For instance, male humpbacks modified their courtship calls in response to sonar exposure [63]. Furthermore, the distance and time intervals between the successive surfacing of humpbacks (*M. novaeangliae*) off North Kauai, Hawaii were found to increase with raised sound levels received from the Acoustic Thermometry of Ocean Climate (ATOC) experiment [65].

The distances within which various marine organisms might be affected by the noise generated by pile driving has been evaluated by Bailey *et al.* [66], who suggested that strong avoidance behavior would only be expected within 20 km of the noise source. A smaller impact zone, within 14 km of pile driving spot for pinnipeds was expected. Though the auditory injury of bottlenose dolphins (*T. truncatus*) would only occur within 100 m of the pile driving location, behavioral disturbance could be expected up to 50 km. Similarly, the behavioral disturbance of minke whales (*Balaenoptera acutorostrata*) may occur as far as 40 km from the pile driving location [66].

Only a few studies have found little or no impact of a given anthropogenic noise on some aspects of some marine organisms, which is probably due to the different characteristics in the received noise and its effects on various species. For example, Wardle found that neither fish nor invertebrates migrated from the reef after noise exposure generated by a seismic triple G. air-gun (three synchronized airguns, each gun 2.51 and 2000 psi), which is probably due to the fact that leaving the habitat costs more than noise exposure for those reef species [67].

**Table 3:** Example studies showing effects of anthropogenic noise on the individual behavior of marine organisms.

Species	Types of Anthropogenic Noise	Effects	References
*D. labrax*	experimental noise emanating device	induces startle response	Kastelein *et al.*, 2008 [6]
*C. labrosus*			
*T. luscus*			
*G. morhua*			
*P. pollachius*			
*T. trachurus*			
*A. Anguilla*			
*C. harengus*			
*P. dentex*	seismic air-gun shooting	induces startle response	Fewtrell and McCauley, 2012 [54]
*P. auratus*			
*S. australis*			
*C. pallasii*	boating and shipping noise	induces avoidance responses	Schwarz and Greer, 1984 [38]
*N. pulcher*	boating and shipping noise	reduces digging and defense capabilities, increases aggression	Bruintjes and Radford, 2013 [58]
*G. aculeatus*	experimental noise emanating device	increases in food-handling error	Purser and Radford, 2011 [59]
*C. clypeatus*	boating and shipping noise	reduces defense capabilities	Chan *et al.*, 2010 [60]
*C. maenas*	boating and shipping noise	reduces defense capabilities	Wale *et al.*, 2013 [61]
*M. novaeangliae*	ATOC (Acoustic Thermometry of Ocean Climate) sound	increases distance and time intervals between successive surfacing	Frankel and Clark, 2000 [65]
*M. novaeangliae*	Sonar	modifies courtship calls	Miller, 2000 [63]
*T. truncatus*	pile driving noise	modifies sound producing	David, 2006 [62]
*E. glacialis*	vessels noise	modifies calling behavior	Parks *et al.*, 2007 [64]
*E. australis*			
*G. cruentatus*	boating and shipping noise	decreases time in nest caring and increases time in the shelters	Picciulin *et al.*, 2010 [57]
*C. chromis*			
*C. caretta*	seismic air-gun shooting	induces startle response	DeRuiter *et al.*, 2012 [56]
*M. densirostris*	mid-frequency sonar	disrupts foraging and induces avoidance behavior	Tyack *et al.*, 2011 [55]

### 3.3. Changes in Population Distribution and Abundance

The induced emigration, unbalanced prey-predator relationship, and reduced recruitment caused by the hampering of embryogenesis by noise exposure have a huge impact on the regional population structure (Table 4). In addition, the relationships between the anthropogenic noise and mass strandings of Cuvier’s beaked whales (*Ziphius cavirostris*) and giant squid (*A. dux*) may represent extreme examples of how anthropogenic noise is reshaping the population distribution and abundance of marine species [53,68]. Similar results were found with regard to Kemp’s ridley sea turtles (*Lepidochelys kempii*) and bottlenose dolphins (*Tursiops truncates*), whose mass strandings were recorded on beaches in the northwestern Gulf of Mexico when explosives were used to remove several oil platforms in adjacent offshore waters [69]. Similarly, the strandings of Cuvier’s beaked whale (*Ziphius cavirostris*), Blainville’s beaked whale (*M. densirostris*), Gervais’ beaked whale (*M. europaeus*) often occurred after the onset of midfrequency sonar by international naval exercises were reported. Further investigation revealed that the whales had suffered severe diffuse congestion and hemorrhage, especially around the acoustic jaw fat, ears, brain, and kidneys. Gas bubble-associated lesions and fat embolisms were observed in the vessels and parenchyma of vital organs. It has been inferred that modified diving behavioral responses to acoustic exposure induced the gas-bubble formation and subsequently caused nitrogen supersaturation above the tolerated threshold, which is a plausible mechanism for the morbidity and mortality seen in cetaceans associated with sonar exposure [70,71,72].

In general, free-swimming marine species may leave an unfavorably noisy environment resulting in a reduction in population density. For instance, Morton found that the occurrence of killer whales (*O. orca*) declined significantly in the Broughton Archipelago after the high-amplitude acoustic harassment devices (AHDs) were installed [73]. Similarly, significantly fewer harbor porpoises (*P. phocoena*) and bottlenose dolphins (*T. truncatus*) were reported after the commencement of operation of offshore wind turbines [74,75]. The reported reduction in catch rates is an effect of anthropogenic noise on the local population abundance of marine fishes [36,41,43,76]. Skalski *et al.* showed that the sound emission from a single air-gun in a hook-and-line fishery for rockfish (*Sebastes* spp.) located along the central California coast led to an average 52% decline in catch rates, causing an average economic loss of 49.8% [43]. Similar catch rate reduction effects caused by anthropogenic noise were also found in cod (*G. morhua*) [36,41], haddock (*M. aeglefinus*) [41,42], and saithe (*Pollachius virens*) [40]. The catch rate of rock lobsters (*Panulirus cygnus*) in western Victoria, Australia was not affected by the seismic surveys, which was probably due to the species’ differences in noise tolerance and the reduction in predator avoidance caused by noise [77].

Other than causing horizontal migration, noise can also lead to a vertical population spatial distribution change. The blue whiting (*Micromesistius poutassou*) and mesopelagic species were found to move to deeper waters in the seismic shooting periods, indicating the population’s short-term avoidance of shooting noise [4]. However, a contrary result was reported by Wilhelmsson, who found that the abundance of demersal fish was greater in the vicinity of the turbines than in the surrounding areas, which may be due to the fact that most submerged parts of the turbines were covered by blue mussels and barnacles and, therefore, functioned as artificial reefs and fish aggregation devices for small demersal fish [78].

It has been shown that noise pollution can lead to a reduction in population recruitment of some marine species [35,39,79]. For instance, noise exposure during larval development resulted in an increase in body malformations for scallops (*Pecten novaezelandiae*) [39]. Similarly, the median time to metamorphosis (TTM) for megalopae of the crabs *Austroelice crassa* and *Hemigrapsus crenulatus* was significantly increased by at least 18 h when exposed to either tidal turbine or wind turbine sound, compared to those in silent control treatments. When the two species were subjected to natural habitat sound, the median TTM decreased by 21%–31%, 38%–47%, and 46%–60% compared to those exposed to silent control, the tidal turbine noise, and the wind turbine noise, respectively [80]. Moreover, though exposure to a single discharge of an array of seismic air-guns did not affect the larval survival of one crustacean species, the Dungeness crab (*Metacarcinus magister*) [79], a significant reduction in reproduction rates after noise exposure was detected in another crustacean species, the brown shrimp (*Crangon crangon*) [35].

### 3.4. The Other Physiological Impacts

In addition to causing auditory masking and histological damage in hearing systems leading to behavioral alteration, and changing population distribution and abundance, noise pollution can induce a series of physiological responses (Table 5) [81,82,83,84]. For example, Casper *et al.* investigated the effects of impulsive pile driving noise on two size groups of hybrid striped bass (white bass *Morone chrysops* crossed with striped bass *M. saxatilis*). The results showed that the larger striped bass (mean size 17.2 g) were more susceptible to barotrauma injuries than the smaller fish (mean size 1.3 g) both in terms of the number of individuals affected and the severity of the injury [85].

Noise generally leads to typical physiological stress responses in marine organisms, such as stimulating nervous activity, increasing metabolism, and reducing immunity. In white whales (*Delphinapterus leucas*), the norepinephrine, epinephrine and dopamine levels were found to increase significantly after high-level sound exposures (>100 kPa) from a seismic water gun (impulse peak pressure levels ranged from approximately 8 to 200 kPa or 198–226 dB re 1 μPa peak pressure) compared to low-level sound exposures (<100 kPa) or controls without noise exposure, representing a nervous activation effect of noise exposure [81]. In white whales (*D. leucas*), a physiological activation, tachycardia, caused by ship noise exposure was detected as well [86]. Similarly, in bottlenose dolphins (*T. truncates*), a significant increase in aldosterone and a significant decrease in monocytes were found after exposure to seismic air-gun noise (44–207 kPa or 213–226 dB re 1 μPa peak pressure) [81]. Moreover, a higher metabolic rate was found by Wale *et al.* in the shore crab (*C. maenas*), as individuals consumed more oxygen when exposed to a ship-noise playback than those exposed to ambient-noise playback, indicating potentially greater stress [82]. When disturbed by noise, marine organisms tend to reduce their food intake and increase their metabolic rate, thereby usually displaying a reduction in growth and other morphological effects [35,83]. For instance, Anderson *et al.* found that the lined seahorses (*Hippocampus erectus*) had significantly smaller ΔWt (body weight change) and larger ΔK (Fulton condition factor change) when exposed to loud noise from aquarium systems [83]. Noise exposure also caused a significant increase in metabolism and a reduction in growth rate in brown shrimp (*C. crangon*) [35]. Similarly, cortisol, glucose, and lactate concentrations in serum increased significantly while AMP, ADP and ATP decreased significantly in the European seabass (*D. labrax*) after exposure to acoustic waves generated by an experimental seismic survey air-gun, suggesting a metabolic rate increase under noise stress [84]. Besides the significant increase in lactate and decrease in glucose, haematocrit levels were also found to be affected by vessel traffic noise in the European seabass (*D. labrax*) and the gilthead sea bream (*Sparus aurata*). The haematocrit level is one of most reliable indexes and indicates the level of blood corpuscular components. An increase in haematocrit activity encourages the production of red blood cells for oxygen transport. In both species, significantly higher levels of haematocrit were recorded for the test group as compared with the control group [2].

**Table 4:** Example studies showing effects of anthropogenic noise on the population distribution and abundance of marine organisms.

Species	Types of Anthropogenic Noise	Effects	References
*Z. cavirostris*	Sonar	causes mass strandings	Frantzis, 1998 [68]
*A. dux*	seismic air-gun shooting	causes mass strandings	Guerra *et al.*, 2011 [53]
*O. orca*	high-amplitude acoustic harassment devices	induces emigration	Morton, 2002 [73]
*P. phocoena*	pile driving noise	induces emigration	Thompson *et al.*, 2010 [75]
*T. truncatus*			
*C. harengus*,	seismic air-gun shooting	induces emigration	Slotte *et al.*, 2004 [4]
*M. poutassou*			
*P. phocoena*	wind farm noise	induces emigration and alters vertical distribution	Carstensen *et al.*, 2006 [74]
*G. flavescens*	wind farm noise	no detectable effects on community structure and biodiversity	Wilhelmsson *et al.*, 2006 [78]
*P. minutus*			
*P. microps*			
*T. bubalis*			
*M. scorpius*			
*S. goodie*	seismic air-gun shooting	decreases catch rate	Skalski *et al.*, 1992 [43]; Løkkeborg *et al.*, 1993 [36]; Engås *et al.*, 1996 [41]
*S. paucispinis*			
*S. chlorostictus*			
*G. morhua*			
*M. aeglefinus*			
*P. virens*	boating and shipping noise	decreases catch rate	Engås, 1994 [40]
*M. aeglefinus*	experimental noise emanating device	decreases catch rate	Nicholson *et al.*, 1992 [42]
*P. cygnus*	seismic air-gun shooting	no detectable effect on catch rate	Parry and Gason, 2006 [77]
*P. novaezelandiae*	experimental noise emanating device	decreases population recruitment	Aguilar de Soto *et al.*, 2013 [39]
*A. crassa*	tidal turbine and wind turbine noise	decreases population recruitment	Pine *et al.*, 2012 [80]
*H. crenulatus*			
*C. crangon*	experimental noise emanating device	decreases reproduction rates	Lagardère, 1982 [35]
*M. magister*	seismic air-gun shooting	no detectable effect on larval survival	Pearson *et al.*, 1994 [79]
*Z. cavirostris*	naval sonar	mass strandings	Cox, *et al.*, 2006 [70]
*M. densirostris*			
*M. europaeus*			
*Z. cavirostris*	naval sonar	mass strandings	Fernández, *et al.*, 2005 [71]
*M. densirostris*			
*M. europaeus*			
*Z. cavirostris*	naval sonar	mass strandings	Jepson, *et al.*, 2003 [72]
*M. densirostris*			
*M. europaeus*			
*L. kempii*	Underwater explosives	mass strandings	Klima *et al.*, 1988 [69]
*T. truncates*			
*C. caretta*			

**Table 5:** Example studies showing physiological impacts of anthropogenic noise on marine organisms.

Species	Types of Anthropogenic Noise	Effects	References
*C. crangon*	experimental noise emanating device	increases metabolism and decreases growth	Lagardère, 1982 [35]
*D. leucas*	seismic air-gun shooting	increases metabolism and decreases immunity	Romano *et al.*, 2004 [81]
*T. truncates*			
*H. erectus*	increased ambient noise	increases metabolism and decreases growth and immunity	Anderson *et al.*, 2011 [83]
*C. maenas*	ship noise	increases metabolism	Wale *et al.*, 2013 [82]
*D. labrax*	seismic air-gun shooting	increases metabolism	Santulli *et al.*, 1999 [84]
*D. labrax*	boating and ship noise	increases metabolism and induces motility	Buscaino *et al.*, 2010 [2]
*S. aurata*			
*D. leucas*	experimental noise emanating device	increases heart rate	Lyamin *et al.*, 2011 [86]
*P. elephas*	ship noise	decreases immunity	Celi *et al.*, 2014 [87]
*M. chrysops*	pile driving	induces barotraumas injuries	Casper *et al.*, 2013 [85]
*M. saxatilis*			

A study carried out by Celi *et al.* showed that ship noise exposure led to a significant decrease in the total haemocyte count (THC) and phenoloxidase (PO) activity in cell-free haemolymph, as well as a significant increase in the haemolymphatic protein concentration and heat shock protein 27 (Hsp 27) expression in haemocyte lysate of the European spiny lobster (*Palinurus elephas*), suggesting that noise has impacts on immunity as well [87]. Noise exposure also led to a depression in the immune system in the white whale (*D. leucas*), with a decrease in alkaline phosphatase level and increase in γ-glutamyltransferase level [86]. Similarly, when lined seahorses (*H. erectus*) were exposed in loud noise tanks, heterophils constituted a significantly greater proportion of their leukocyte population and their heterophil:lymphocyte ratio (H:L ratio) was significantly greater than when in quiet tanks. Plasma cortisol concentrations were higher and kidneys were significantly more affected by parasites in loud tanks [83].

## 4. Discussion and Conclusions

With growing utilization and exploration of the ocean by human beings, the impact of anthropogenic noise on marine organisms has become one of the most important research topics. As summarized in the present review, the effects of anthropogenic noise on marine organisms are dependent on the species investigated and both the levels of impulsive and stationary noise. For example, when the same species, *C. harengus*, was exposed to noise at different intensities generated by an experimental noise emanating device, boating and shipping, and seismic air-guns, the response varied from startle and avoidance to forced emigration, respectively [4,6,38]. Similarly, the effects of the same noise on various species were shown to be species-specific due to the intrinsic differences among species. When exposed to the same kind of noise, boating and shipping noise, avoidant behavior, reduction in nest-digging and defense capability, and increased distance and time intervals between successive surfacing were found for *C. harengus*, *N. pulcher*, and *M. novaeangliae* respectively [38,58,65]. An extreme example exhibiting species-specific difference in response to anthropogenic noise even showed that, unlike noise, music enhanced the growth of *S. aurata* [2,88,89]. However, since various kinds of noise, including seismic air-gun noise, boating and shipping noise, sonar, white noise, and noise generated by experimental noise emanating device with wide range of intensities and frequencies, have been studied by different researchers, it is difficult to compare the reported results.

**Figure 1:**
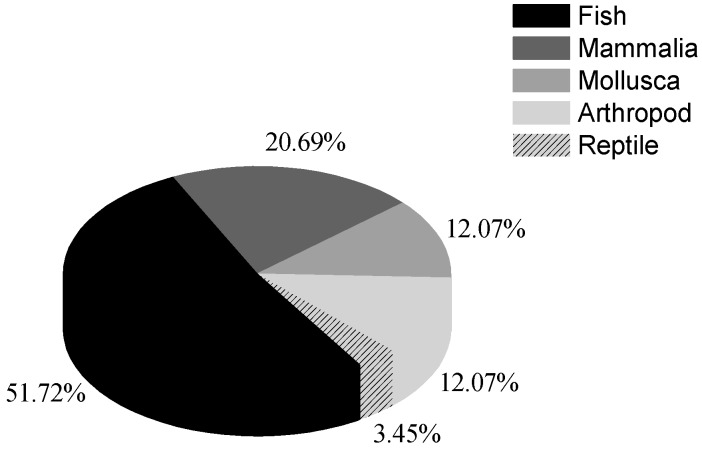
The percentage of studied marine species in different taxa being affected by anthropogenic noise.

Through statistics (Figure 1), most studies investigating the effects of anthropogenic noise on marine organisms were carried out in fish, which account for half of the total number of the species studied with regard to noise-effects. The effects of noise on marine mammalian species, mainly whales and dolphins, were also effectively investigated and account for more than 20% of the total species investigated. Only a few studies have been conducted with invertebrates and reptiles.

Since the effects of anthropogenic noise on marine organisms are species specific and marine organisms at different life stages may show differences in their susceptibilities, the present knowledge of the biological effects of noise is still limited. Previous studies have indicated that individuals in different life stages showed different reactions to various environmental disturbances. To date, most studies have investigated the effects of anthropogenic noise on adults. Therefore, the scarcity of studies conducted with larval and/or juvenile individuals, who are probably more susceptible to anthropogenic noise, severely constrains our present understanding of noise pollution. Furthermore, almost all the studies were carried out with species having typical hearing acoustic receiving structures such as ears and lateral lines due to the obvious logical relevance between noise and acoustic sensory of the organisms. However, the effects of noise on organisms without obvious acoustic receiving structures were largely overlooked. Theoretically, in the far field of an acoustic source, the pressure (*p*) and velocity components (*v*) are related as *p* = *v* × *z*, where *z* is the parameter that indicates the impedance of the medium [39]. Therefore, the particle motion in the water driven by noise may be detected by the body surface of marine organisms directly. For instance, it has been shown by the only study carried out with a bivalve species that the larval development of the scallop *P. novaezelandiae*, which have no reported acoustic receiving structure, was significantly obstructed by the noise exposure [39]. Therefore, the effects of anthropogenic noise on marine organisms with no apparent acoustic receiving structures should be addressed.

It is notable that some studies were conducted with exceedingly small sample size, due to either the low population density of the specimen in the survey area or the low availability of the specimen in the laboratory. The insufficient sample size severely hampers the reliability of the conclusion. Another technical difficulty lies in the evaluation of the impacts of noise isolated from other environmental stressors, such as pollution, climate change, and ocean acidification. Conducting the investigation in the laboratory seems to be a good solution to isolate noise from other environmental stressors. However, the results obtained fail to represent the real natural scenario. Fully understandings of the impacts of noise on marine organisms in the sea need not only investigating the biological responses of the species but also comprehensive information about the noise in the sea such as sound levels, the distance between species and sound source, the propagation loss, as well as ambient sound condition. Therefore, detailed description about the type, levels, and frequencies of sound studied is necessary, and is something that has been skipped by some studies.

At present, why and how noise affects marine organisms are still poorly understood, with only some aspects and an extremely small proportion of the marine organisms having been investigated. The answers to many questions remain unknown, such as “is the noise signal been perceived by marine bivalves as a common environmental stress or informative messages about the surrounding environment?” and “can sessile marine species adapt to non-lethal noise environments?”

## References

[1] Andrew R.K., Howe B.M., Mercer J.A., Dzieciuch M.A. (2002). Ocean ambient sound: Comparing the 1960s with the 1990s for a receiver off the California coast. Acoust. Res. Lett..

[2] Buscaino G., Filiciotto F., Buffa G., Bellante A., di Stefano V., Assenza A., Fazio F., Caola G., Mazzola S. (2010). Impact of an acoustic stimulus on the motility and blood parameters of European sea bass (*Dicentrarchus labrax* L.) and gilthead sea bream (*Sparus aurata* L.). Mar. Environ. Res..

[3] Johansson K. (2011). Impact of Anthropogenic Noise on Fish Behaviour and Ecology. http://pub.epsilon.slu.se/8366/1/Johansson_K_111013.pdf.

[4] Slotte A., Hansen K., Dalen J., Ona E. (2004). Acoustic mapping of pelagic fish distribution and abundance in relation to a seismic shooting area off the Norwegian west coast. Fish. Res..

[5] Tyack P.L. (2008). Implications for marine mammals of large-scale changes in the marine acoustic environment. J. Mammal..

[6] Kastelein R.A., Heul S., Verboom W.C., Jennings N., Veen J., de Haan D. (2008). Startle response of captive North Sea fish species to underwater tones between 0.1 and 64 kHz. Mar. Environ. Res..

[7] Codarin A., Wysocki L.E., Ladich F., Picciulin M. (2009). Effects of ambient and boat noise on hearing and communication in three fish species living in a marine protected area (Miramare, Italy). Mar. Pollut. Bull..

[8] André M. (2009). The sperm whale sonar: Monitoring and use in mitigation of anthropogenic noise effects in the marine environment. Nucl. Instrum. Methods Phys. Res. Sect. A.

[9] Kight C.R., Swaddle J.P. (2011). How and why environmental noise impacts animals: An integrative, mechanistic review. Ecol. Lett..

[10] Popper A.N., Hastings M.C. (2009). The effects of human-generated sound on fish. Integr. Zool..

[11] Slabbekoorn H., Bouton N., van Opzeeland I., Coers A., Cate C., Popper A.N. (2010). A noisy spring: The impact of globally rising underwater sound levels on fish. Trends Ecol. Evolut..

[12] Conservation and Development Problem Solving Team (2000). Anthropogenic Noise in the Marine Environment: Potential Impacts on the Marine Resources and Stellwagen Bank and Channel Islands National Marine Sanctuaries.

[13] Rogers P.H., Cox H., Atema J., Fay R.R., Popper A.N. (1988). Underwater sound as a biological stimulus. Sensory Biology of Aquatic Animals.

[14] Pitcher T.J. (1992). Behaviour of Teleost Fishes.

[15] Simmonds J., MacLennan D.N. (2005). Fisheries Acoustics: Theory and Practice.

[16] Helfman G., Collette B.B., Facey D.E., Bowen B.W. (2009). The Diversity of Fishes: Biology, Evolution and Ecology.

[17] Hawkins A., Rasmussen K.J. (1978). The calls of gadoid fish. J. Mar. Biol. Assoc. UK.

[18] Everest F.A., Young R.W., Johnson M.W. (2005). Acoustical characteristics of noise produced by snapping shrimp. J. Acoust. Soc. Am..

[19] Ladich F., Yan H. (1998). Correlation between auditory sensitivity and vocalization in anabantoid fishes. J. Comp. Physiol. A.

[20] Popper A.N., Fay R.R., Platt C., Sand O., Collins S.P., Marshall N.J. (2003). Sound detection mechanisms and capabilities of teleost fishes. Sensory Processing in Aquatic Environments.

[21] Urick R.J. (1983). Principles of Underwater Sound.

[22] McDonald M.A., Hildebrand J.A., Wiggins S.M. (2006). Increases in deep ocean ambient noise in the Northeast Pacific west of San Nicolas Island, California. J. Acoust. Soc. Am..

[23] Ross D. (2005). Ship sources of ambient noise. IEEE J. Ocean. Eng..

[24] Buck E.H. (1995). Acoustic Thermometry of Ocean Climate: Marine Mammal Issues.

[25] Gisiner R., Cudahy E., Frisk G., Gentry R., Hofman R., Popper A., Richardson W.J. (1998). Proceedings: Workshop on the Effects of Anthropogenic Noise in the Marine Environment.

[26] Richardson W.J., Finley K.J., Miller G.W., Davis R.A., Koski W.R. (1995). Feeding, social and migration behavior of bowhead whales, *Balaena mysticetus*, in Baffin Bay *vs.* the Beaufort Sea—Regions with different amount of human activity. Mar. Mammal Sci..

[27] Simard Y., Lepage R., Gervaise C. (2010). Anthropogenic sound exposure of marine mammals from seaways: Estimates for Lower St. Lawrence Seaway, eastern Canada. Appl. Acoust..

[28] Amoser S., Wysocki L.E., Ladich F. (2004). Noise emission during the first powerboat race in an Alpine lake and potential impact on fish communities. J. Acoust. Soc. Am..

[29] Bregman A.S. (1994). Auditory Scene Analysis: The Perceptual Organization of Sound.

[30] Popper A.N., Hastings M.C. (2009). The effects of anthropogenic sources of sound on fishes. J. Fish Biol..

[31] Southall B.L., Bowles A.E., Ellison W.T., Finneran J.J., Gentry R.L., Greene C.R., Kastak D., Ketten D.R., Miller J.H., Nachtigall P.E. (2008). Marine mammal noise-exposure criteria: Initial scientific recommendations. Bioacoustics.

[32] Brawn V.M. (1961). Sound production by the cod (*Gadus callarias* L.). Behaviour.

[33] Schwarz A.L. (1985). The behavior of fishes in their acoustic environment. Environ. Biol. Fishes.

[34] Scholik A.R., Yan H.Y. (2002). The effects of noise on the auditory sensitivity of the bluegill sunfish, *Lepomis macrochirus*. Comp. Biochem. Physiol. Part A.

[35] Lagardère J. (1982). Effects of noise on growth and reproduction of *Crangon crangon* in rearing tanks. Mar. Biol..

[36] Løkkeborg S., Soldal A.V. (1993). The influence of seismic exploration with airguns on cod (*Gadus morhua*) behaviour and catch rates. ICES J. Mar. Sci. Symp..

[37] McCauley R.D., Fewtrell J., Popper A.N. (2003). High intensity anthropogenic sound damages fish ears. J. Acoust. Soc. Am..

[38] Schwarz A.L., Greer G.L. (1984). Responses of Pacific herring, *Clupea harengus pallasi*, to some underwater sounds. Can. J. Fish. Aquat. Sci..

[39] Aguilar de Soto N., Delorme N., Atkins J., Howard S., Williams J., Johnson M. (2013). Anthropogenic noise causes body malformations and delays development in marine larvae. Sci. Rep..

[40] Engås A., Ferno A., Olsen S. (1994). The effects of trawl performance and fish behaviour on the catching efficiency of demersal sampling trawls. Marine Fish Behaviour in Capture and Abundance Estimation.

[41] Engås A., Løkkeborg S., Ona E., Soldal A.V. (1996). Effects of seismic shooting on local abundance and catch rates of cod (*Gadus morhua*) and haddock (*Melanogrammus aeglefinus*). Can. J. Fish. Aquat. Sci..

[42] Nicholson M., Rackham B., Mitson R. Measuring the effect of underwater radiated noise on trawl catches. Proceedings of the ICES FTFB and FAST Joint Working Group Meeting.

[43] Skalski J.R., Pearson W.H., Malme C.I. (1992). Effects of sounds from a geophysical survey device on catch-per-unit-effort in a hook-and-line fishery for rockfish (*Sebastes* spp.). Can. J. Fish. Aquat. Sci..

[44] Clark C.W., Ellison W.T., Southall B.L., Hatch L., Parijs S.M., Frankel A., Ponirakis D. (2009). Acoustic masking in marine ecosystems: Intuitions, analysis, and implication. Mar. Ecol. Prog. Ser..

[45] Southall B.L., Schusterman R.J., Kastak D. (2003). Acoustic communication ranges for northern elephant seals (*Mirounga angustirostris*). Aquat. Mammal..

[46] Vasconcelos R.O., Amorim M.C., Ladich F. (2007). Effects of ship noise on the detectability of communication signals in the Lusitanian toadfish. J. Exp. Biol..

[47] Aguilar de Soto N., Johnson M., Madsen P.T.M., Tyack P.L., Bocconcelli A., Borsani F. (2006). Dose intense ship noise disrupt foraging in deep-diving Cuvier’s beaked whales (*Ziphius cavirostris*)?. Mar. Mammal Sci..

[48] Lucke K., Siebert U., Lepper P.A., Blanchet M.A. (2009). Temporary shift in masked hearing thresholds in a harbor porpoise (*Phocoena phocoena*) after exposure to seismic airgun stimuli. J. Acoust. Soc. Am..

[49] Nachtigall P.E., Supin A.Y., Pawloski J., Au W.W. (2004). Temporary threshold shifts after noise exposure in the bottlenose dolphin (*Tursiops truncatus*) measured using evoked auditory potentials. Mar. Mammal Sci..

[50] Popper A.N., Smith M.E., Cott P.A., Hanna B.W., MacGillivray A.O., Austin M.E., Mann D.A. (2005). Effects of exposure to seismic airgun use on hearing of three fish species. J. Acoust. Soc. Am..

[51] Scholik A.R., Yan H.Y. (2001). Effects of underwater noise on auditory sensitivity of a cyprinid fish. Hear. Res..

[52] André M., Solé M., Lenoir M., Durfort M., Quero C., Mas A., Lombarte A., van der Schaar M., López-Bejar M., Morell M. (2011). Low-frequency sounds induce acoustic trauma in cephalopods. Front. Ecol. Environ..

[53] Guerra Á., González Á.F., Pascual S., Dawe E.G. (2011). The giant squid *Architeuthis*: An emblematic invertebrate that can represent concern for the conservation of marine biodiversity. Biol. Conserv..

[54] Fewtrell J.L., McCauley R.D. (2012). Impact of air gun noise on the behaviour of marine fish and squid. Mar. Pollut. Bull..

[55] Tyack P.L., Zimmer W.M., Moretti D., Southall B.L., Claridge D.E., Durban J.W., Clark C.W., D’Amico A., DiMarzio N., Jarvis S. (2011). Beaked whales respond to simulated and actual navy sonar. PLoS ONE.

[56] DeRuiter S., Larbi Doukare K. (2012). Loggerhead turtles dive in response to airgun sound exposure. Endanger. Species Res..

[57] Picciulin M., Sebastianutto L., Codarin A., Farina A., Ferrero E.A. (2010). *In situ* behavioural responses to boat noise exposure of *Gobius cruentatus* (Gmelin, 1789; fam. Gobiidae) and *Chromis chromis* (Linnaeus, 1758; fam. Pomacentridae) living in a Marine Protected Area. J. Exp. Mar. Biol. Ecol..

[58] Bruintjes R., Radford A.N. (2013). Context-dependent impacts of anthropogenic noise on individual and social behaviour in a cooperatively breeding fish. Anim. Behav..

[59] Purser J., Radford A.N. (2011). Acoustic noise induces attention shifts and reduces foraging performance in three-spined sticklebacks (*Gasterosteus aculeatus*). PLoS ONE.

[60] Chan A.A., Giraldo-Perez P., Smith S., Blumstein D.T. (2010). Anthropogenic noise affects risk assessment and attention: The distracted prey hypothesis. Biol. Lett..

[61] Wale M.A., Simpson S.D., Radford A.N. (2013). Noise negatively affects foraging and antipredator behaviour in shore crabs. Anim. Behav..

[62] David J.A. (2006). Likely sensitivity of bottlenose dolphins to pile-driving noise. Water Environ. J..

[63] Miller P.J., Biassoni N., Samuels A., Tyack P.L. (2000). Whale songs lengthen in response to sonar. Nature.

[64] Parks S.E., Clark C.W., Tyack P.L. (2007). Short- and long-term changes in right whale calling behavior: The potential effects of noise on acoustic communication. J. Acoust. Soc. Am..

[65] Frankel A., Clark C. (2000). Behavioral responses of humpback whales (*Megaptera novaeangliae*) to full-scale ATOC signals. J. Acoust. Soc. Am..

[66] Bailey H., Senior B., Simmons D., Rusin J., Picken G., Thompson P.M. (2010). Assessing underwater noise levels during pile-driving at an offshore windfarm and its potential effects on marine mammals. Mar. Pollut. Bull..

[67] Wardle C., Carter T., Urquhart G., Johnstone A., Ziolkowski A., Hampson G., Mackie D. (2001). Effects of seismic air guns on marine fish. Cont. Shelf Res..

[68] Frantzis A. (1998). Does acoustic testing strand whales?. Nature.

[69] Klima E.F., Gitschlag G.R., Renaud M.L. (1988). Impacts of the explosive removal of offshore petroleum on sea turtles and dolphins. Mar. Fish. Rev..

[70] Cox T.M., Ragen T.J., Read A.J., Vos E., Baird R.W., Balcomb K., Barlow J., Caldwell J., Cranford T., Crum L. (2006). Understanding the impacts of anthropogenic sound on beaked whales. J. Cetacean Res. Manag..

[71] Fernández A., Edwards J.F., Rodríguez F., Espinosa de los Monteros A., Herráez P., Castro P., Jaber J.R., Martín V., Arbelo M. (2005). “Gas and fat embolic syndrome” involving a mass stranding of beaked whales (family *Ziphiidae*) exposed to anthropogenic sonar signals. Vet. Pathol..

[72] Jepson P.D., Arbelo M., Deaville R., Patterson I.A.P., Castro P., Baker J.R., Degollada E., Ross H.M., Herráez P., Pocknell A.M. (2003). Gas-bubble lesions in stranded cetaceans: Was sonar responsible for a spate of whale deaths after an Atlantic military exercise?. Nature.

[73] Morton A. (2002). Displacement of *Orcinus orca* (L.) by high amplitude sound in British Columbia, Canada. ICES J. Mar. Sci..

[74] Carstensen J., Henriksen O.D., Teilmann J. (2006). Impacts of offshore wind farm construction on harbour porpoises: Acoustic monitoring of echolocation activity using porpoise detectors (T-PODs). Mar. Ecol. Prog. Ser..

[75] Thompson P.M., Lusseau D., Barton T., Simmons D., Rusin J., Bailey H. (2010). Assessing the responses of coastal cetaceans to the construction of offshore wind turbines. Mar. Pollut. Bull..

[76] Engås A., Løkkeborg S. (2002). Effects of seismic shooting and vessel-generated noise on fish behaviour and catch rates. Bioacoustics.

[77] Parry G.D., Gason A. (2006). The effect of seismic surveys on catch rates of rock lobsters in western Victoria, Australia. Fish. Res..

[78] Wilhelmsson D., Malm T., Ohman M. (2006). The influence of offshore windpower on demersal fish. ICES J. Mar. Sci..

[79] Pearson W.H., Skalski J.R., Sulkin S.D., Malme C.I. (1994). Effects of seismic energy releases on the survival and development of zoeal larvae of dungeness crab (*Cancer magister*). Mar. Environ. Res..

[80] Pine M.K., Jeffs A.G., Radford C.A. (2012). Turbine sound may influence the metamorphosis behaviour of estuarine crab megalopae. PLoS ONE.

[81] Romano T.A., Keogh M.J., Kelly C., Feng P., Berk L., Schlundt C.E., Carder D.A., Finneran J.J. (2004). Anthropogenic sound and marine mammal health: Measures of the nervous and immune systems before and after intense sound exposure. Can. J. Fish. Aquat. Sci..

[82] Wale M.A., Simpson S.D., Radford A.N. (2013). Size-dependent physiological responses of shore crabs to single and repeated playback of ship noise. Biol. Lett..

[83] Anderson P.A., Berzins I.K., Fogarty F., Hamlin H.J., Guillette L.J. (2011). Sound, stress, and seahorses: The consequences of a noisy environment to animal health. Aquaculture.

[84] Santulli A., Modica A., Messina C., Ceffa L., Curatolo A., Rivas G., Fabi G., D’amelio V. (1999). Biochemical responses of European sea bass (*Dicentrarchus labrax* L.) to the stress induced by off shore experimental seismic prospecting. Mar. Pollut. Bull..

[85] Casper B.M., Halvorsen M.B., Matthews F., Carlson T.J., Pooper A.N. (2013). Recovery of barotraumas injuries resulting from exposure to pile driving sound in two sizes of hybrid striped bass. PLoS ONE.

[86] Lyamin O.I., Korneva S.M., Rozhnov V.V., Mukhametov L.M. (2011). Cardiorespiratory changes in beluga in response to acoustic noise. Dokl. Biol. Sci..

[87] Celi M., Filiciotto F., Vazzana M., Arizza V., Maccarrone V., Ceraulo M., Mazzola S., Buscaino G. (2014). Shipping noise affecting immune responses of European spiny lobster (*Palinurus elephas*). Can. J. Zool..

[88] Filiciotto F., Giacalone V.M., Fazio F., Buffa G., Piccione G., Maccarrone V., di Stefano V., Mazzola S., Buscaino G. (2013). Effect of acoustic environment on gilthead sea bream (*Sparus aurata*): Sea and onshore aquaculture background noise. Aquaculture.

[89] Papoutsoglou S.E., Karakatsouli N., Batzina A., Papoutsoglou E.S., Tsopelakos A. (2008). Effect of music stimulus on gilthead seabream *Sparus aurata* physiology under different light intensity in a re-circulating water system. J. Fish Biol..

